# Effect of different mixing and placement methods on the quality of MTA apical plug in simulated apexification model

**DOI:** 10.4317/jced.53410

**Published:** 2017-03-01

**Authors:** Negin Ghasemi, Maryam Janani, Tahmineh Razi, Faezeh Atharmoghaddam

**Affiliations:** 1 Assistant Professor, Department of Endodontics, Dental and Periodontal Research Center, Dental Faculty, Tabriz University (Medical Sciences), Tabriz, Iran; 2 Assistant Professor, Department of Endodontics, Dental Faculty, Tabriz University (Medical Sciences), Tabriz, Iran; 3 Assistant Professor, Department of Radiology, Dental Faculty, Tabriz University (Medical Sciences), Tabriz, Iran; 4 Student of Dentistry, Dental Faculty, Tabriz University, (Medical Sciences), Tabriz, Iran

## Abstract

**Background:**

It is necessary apical plug material to exhibit proper adaptation with the root canal walls. Presence of voids at the interface between the root canal wall and this material result in micro leakage, which might have a relationship with post treatment disease. The aim of the present study was to evaluate the effect of different mixing (manual and ultrasonic) and placement (manual and manual in association with indirect ultrasonic) method of Mineral Trioxide Aggregate (MTA) on the void count and dimension in the apical plug in natural teeth with simulated open apices.

**Material and Methods:**

Eighty human maxillary central incisors were selected. After simulation of the open apex model, the teeth were assigned to 4 groups based on the mixing and placement techniques of MTA: group 1, manual mixing and manual placement; group 2, manual mixing and manual placement in association with indirect ultrasonic; group 3, ultrasonic mixing and and manual placement; and group 4, ultrasonic mixing and manual placement in association with indirect ultrasonic. The prepared samples were placed within gypsum sockets in which the periodontal ligament was reconstructed with polyether impression material. In group 1, after mixing, the material was condensed with a hand plugger. In group 2, after mixing, the ultrasonic tip was contacted with the hand plugger for 2 seconds. In groups 3 and 4, mixing was carried out with the ultrasonic tip for 5 seconds and in groups 3 and 4, similar to groups 1 and 2, respectively, the materials were placed as apical plugs, measuring 3 mm in length. A wet cotton pellet was placed at canal orifices and dressed with Cavit. After one week, the cone beam computed tomography (CBCT) technique was used to count the number of voids between the material and root canal walls. The void dimensions were determined using the following scoring system: score 1, absence of voids; score 2, the void size less than half of the dimensions of the evaluated cross-section; score 3, the void size larger than half of the dimensions of the evaluated cross-section. Chi-squared and Fisher’s exact tests were used for statistical analyses. Statistical significance was set at *P*<0.05.

**Results:**

The maximum (13) and minimum (3) number of voids were detected in groups 2 and 3, respectively. There were no significant differences between groups 1 and 3 in the number of voids (*p* >0.05). Evaluation of void dimensions showed no score 3 in any of the study groups and the dimensions of all the voids conformed to score 2.

**Conclusions:**

Under the limitations of the present study, use of ultrasonic mixing and manual placement techniques resulted in a decrease in the number of voids in the apical plug.

** Key words:**Apical plug, MTA, ultrasonic, void.

## Introduction

MTA has several uses in endodontics, including its use as an apical plug in the apexification procedure of immature permanent teeth (1). A material used as an apical plug should be able to provide a proper seal in addition to properties such as setting in the presence of blood and moisture, tissue tolerance and antimicrobial activity (2,3). In other words, it is necessary for such a material to exhibit proper adaptation with the root canal walls in order to prevent leakage of bacteria and their products into the periapical tissues (4). Presence of voids at the interface between the root canal wall and the material used to create an apical seal results in the entrapment of toxins and microorganisms, which might have a relationship with post treatment disease (5,6).

MTA is a technique-sensitive material and requires careful delivery to create a proper seal (7). Previous studies in relation to the effect of ultrasonic placement on the quality of MTA plug showed that the effect of ultrasonic technique on improving bacterial seal was not significant; however, it resulted in a denser obturation from a radiographic point of view due to an increase in the effective flow of MTA (8). In addition, indirect activation with an ultrasonic tip in association with manual condensation resulted in a denser material compared to manual condensation alone (9). However, another study showed that manual condensation resulted in better adaptation and less porosity compared to direct ultrasonic technique (10).

Powder-to-liquid ratio and porosity might affect the mechanical properties of dental cements. Therefore, the mixing and placement variables are key factors that affect the performance of dental materials (11,12). Evaluation of previous investigations regarding the effect of mixing technique on various properties of MTA showed that ultrasonic mixing increases its flow and solubility and decreases its setting and working times (13-15).

Identification of voids is important clinically and evaluation of voids between the root canal wall and the material is an acceptable technique for the evaluation of the obturation quality (5,16). Radiography is one of the techniques used for such evaluation; however, analog and digital radiographic techniques have limitations because they yield two-dimensional images of three-dimensional structures, resulting in the superimposition of anatomic structures. In recent years, CBCT technique has been introduced as a non-invasive technique for the evaluation of dentoalveolar structures. The technique uses low radiation dose and has high resolution (17,18). On the other hand, it yields a three-dimensional image and has no geometric distortion problem (19).

No studies to date have been carried out to simultaneously evaluate the effects of different mixing and placing techniques on the quality of the apical plug. The present study was designed to measure the voids in the apical plug with the use of the CBCT technique by considering the above parameters.

## Material and Methods

The protocol of this study was approved by the Ethics Committee of Tabriz University of Medical Sciences (IR.TBZMED.REC.1395.31). A total of 80 human maxillary central incisors with straight and fully developed roots were selected. The teeth had no cracks and fractures. The tooth crowns were removed at the level of cement-enamel junction with the use of a diamond saw (SP 1600 Microtome, Leica, Nu Block, Germany) under water coolant to leave 12 mm of the root length. Then 3 mm of the root was cut away from the apical end to leave 9 mm of the root length in all the samples.

-Simulation of periodontal ligament 

The roots were covered with molten wax and mounted in gypsum blocks. After complete setting of the gypsum (Moldano blue™, Heraues Kulzer, Hanau, Germany), the wax was removed by rinsing with hot water, which resulted in the creation of a gypsum socket. Polyether impression material (Impregum Soft, 3M ESPE, Seefeld, Germany) was poured within the socket and the roots were placed within the socket. After setting of the polyether impression material, the roots were removed from the sockets.

-Root canal preparation and simulation of open apex model

To simulate apexification, the root canals were instrumented using #5, #4, #3, #2 and #1 Gates-Glidden drills (Dentsply Maillefer, Ballaigues, Switzerland) with the crown-down technique. To this end, #5 drill was inserted into the root canal up to 3 mm, followed by 5, 7 and 9 mm of penetration into the root canal by #4, #3 and #2 drills. To simulate the open apex condition, #1 drill was used 1 mm beyond the apical foramen by moving it through the apical foramen once without any resistance. Then #40 RaCe rotary file (FKG, Lachaux-de-Fonds, Switzerland) with 10% taper was inserted into the root canal through the apical foramen so that its entire cutting length was placed in the root canal. A total of 5 mL of 0.5% NaOCl solution was used as a root canal irrigant during instrumentation. At the end of instrumentation, 5 mL of 17% EDTA was used to remove the smear layer. A total of 5 mL of normal saline solution was used as a final irrigant.

-Placement of the apical plug

The samples were randomly divided into 4 groups (n=20) based on the MTA (Angelus, Londrina, Paraná, Brazil) mixing and placement techniques as follows:

Group 1: manual mixing and manual placement

Group 2: manual mixing and manual placement in association with indirect ultrasonic 

Group 3: ultrasonic mixing and manual placement

Group 4: ultrasonic mixing and manual placement in association with indirect ultrasonic 

In all the study groups, MTA was mixed according to manufacturer’s instructions at a powder-to-liquid ratio of 3:1. In group 1, after mixing the material, an MTA carrier was used to carry the material incrementally into the root canal; the material was packed with the use of a hand plugger up to the proper length, which continued until a plug length of 3 mm was achieved. In group 2, a similar approach was used except for the fact that after packing the material with a hand plugger the ultrasonic tip was placed in contact with the plugger for 2 seconds to apply an indirect vibratory motion to the material. The power of the ultrasonic unit was adjusted at its lowest settings. In groups 3 and 4, the material was mixed with the ultrasonic tip for 5 seconds and then the placement was done in group 3, similar to group 1, and in group 4, similar to group 2. A wet cotton pellet was placed at the orifice of all the root canals and the samples were dressed with Cavit (DeTrey/ Dentsply, Konstanz, Germany). Then the samples were incubated at 37°C and 100% relative humidity for 1 week.

-Evaluation of the samples with the CBCT technique

Each root underwent a CBCT imaging procedure with the use of a Newtom VGI unit (Verona QR, Italy) at axial and cross-sectional views by a radiologist. This x-ray unit delivers a conical x-ray beam and has a flat-panel detector, with 1536×1920 and 127×127 pixel sizes, a pixel depth of 14 bits, a rotation of 360°, a scan time of 18 seconds and a KVP of 110. The initial and final reconstructions were carried out with NNT Viewer software program version 2.17. The unit’s exposure conditions were adjusted automatically. Data collected from the CBCT evaluations were entered into the NNT Viewer software program version 2.17. The resultant images were displayed on a 19-inch LCD monitor (PHILIPS, 19013) with a resolution of 1024×1208 pixels and 32 bits in a dimlylit room. The number of voids between the material and the root canal wall was counted in all the samples and recorded as the number of voids in 20 samples in each group. The following scoring system was used to characterize the dimensions of the voids:

Score 1: No voids

Score 2: The size of the void less than half of the dimensions of the cross-section evaluated

Score 3: The size of the void larger than half of the dimensions of the cross-section evaluated

-Statistical analysis

After drawing cross tabs for the data, chi-squared and Fisher’s exact tests were used for statistical analysis of data. The significant difference was set at *p*<0.05.

## Results

 Table 1 presents the number and dimensions of the voids in the study groups. The maximum [13] and minimum [3] number of voids were detected in groups 2 and 3, respectively. The differences between these two groups (*P*=0.001) and between these two groups and groups 1 and 4 were significant (*P*<0.05). There was no significant difference between groups 1 and 3 in the number of voids (*P*=0.7). Evaluation of the dimension of the voids showed no score 3 in any of the study groups and the dimensions of all the voids conformed to score 2.

**Table 1:**
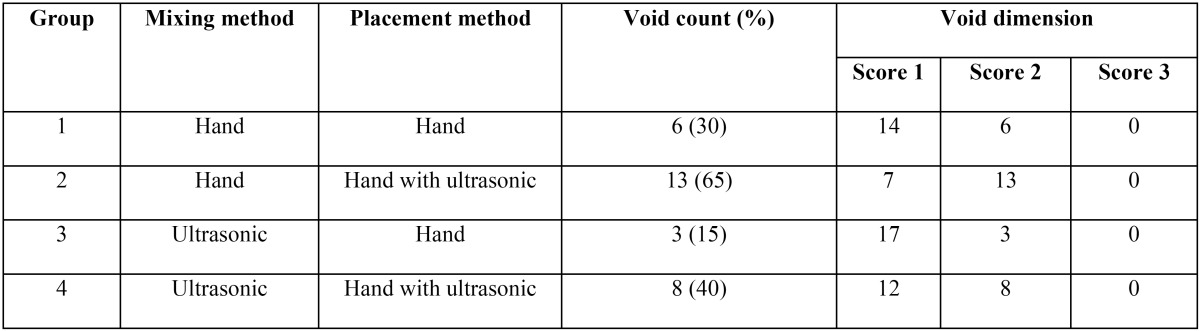
Void counts and dimensions in the study groups.

## Discussion

The present study was undertaken to evaluate the quality of MTA apical plug and the effect of different mixing and placement techniques on the number and dimensions of voids between the material and root canal wall in the apical plug with the use of the CBCT technique. The results showed that in two groups with identical mixing technique, use of manual condensation in association with indirect ultrasonic technique resulted in more voids; on the other hand in two groups with identical placement technique, mixing with an ultrasonic tip resulted in less voids.

MTA is used as an apical in the treatment of permanent immature teeth due to its favorable properties; however, its difficult handling has always been reported as one of its chief disadvantages (20). On the other hand, considering the limited visibility and access at the root end, it is expected that voids will form between the material and root canal walls when it is placed in that area. The presence of voids in the obturation material is considered one of the factors compromising the treatment results; in other words, voids provide a path for the leakage of microorganisms and their products and also a space for the proliferation of microorganisms, paving the way for post treatment disease (5). In other words, the presence of voids between the material and the root canal wall affects the sealing ability of the material. The set mixture of MTA has a honeycomb internal structure, with air pockets that comprise 1-30% of its volume. To improve this situation and increase the density and homogeneity of the resultant mixture, one of the techniques is to use vibration during placement and packing of the material; vibrations create compressive impulses that rearrange the particles and create a denser mass (9).

The effect of ultrasonic technique on the quality of MTA obturation has been evaluated in two studies (9,10). In one study, the ultrasonic energy was directly used during packing of MTA for 30 seconds (10). In that study, MTA was packed in polyethylene tubes and the voids were evaluated with radiographic and light microscopic techniques. The ultrasonic energy in that study increased bubble formation, consistent with the results of the present study. It should be pointed out that there were some differences between the present study and that study. In the present study, natural teeth with an open apex model were used for evaluations to make a better simulation of the clinical situation. In the present study the ultrasonic technique was used indirectly and the application time was shorter than that in the study above. Under the clinical conditions, even the thinnest ultrasonic tips reach the root canal end with difficulty. Therefore, it is very difficult to use such technique directly (9). In addition, previous studies have shown that there are higher odds for bubble formation with an increase in the time and energy of ultrasonic technique and there is low adaptation with the root canal walls. In addition, in the present study, CBCT technique was used for evaluations, making it possible to carry out more accurate evaluations with 0.3-mm accuracy in different cross-sections.

In another study, Yeung *et al.* (9) evaluated the density of MTA with the indirect application of ultrasonic energy in association with manual condensation, in a manner similar to the technique used in the present study. The only difference was that the application time of the ultrasonic energy that was shorter than that in the present study and was 1 second. The results of that study showed that the indirect application of ultrasonic energy resulted in a denser obturation compared to the manual technique alone, with less voids. The technique used for evaluation in that study was different from that in this study because in the present study only superficial voids were evaluated. Vibration with an ultrasonic device results in rearrangement of particles and movement of the voids toward the material’s surface. On the other hand, that study was carried out in root canals with closed apices in acrylic blocks, with no open apex model similar to the one in the present study. It is very difficult to pack MTA in a tooth with an open apex compared to packing it in a tooth with a closed apex with a stop and the odds of bubble formation are higher in teeth with open apices.

The innovation in the present study, compared to the two studies mentioned above, was the use of ultrasonic mixing. In a study carried out by Shahi *et al.*, mixing with the ultrasonic technique resulted in an improvement in some of the properties of MTA, including a decrease in setting time (13). In addition, it has been reported that mixing with the ultrasonic technique results in an increase in the flow of MTA (15), which might explain a decrease in the number of voids compared to the mix achieved after manual mixing.

It is suggested that such a study be carried out in curved canals because it is much more difficult to place MTA at the apical end of curved canals compared to that in straight canals. In addition, it appears it is necessary to design a study to evaluate the density of obturations by weighing the samples. Under the limitations of the present study, it is suggested that ultrasonic mixing and hand placement techniques be used to improve the quality of MTA apical plugs.
